# Severe malaria in children leads to a significant impairment of transitory otoacoustic emissions - a prospective multicenter cohort study

**DOI:** 10.1186/s12916-015-0366-8

**Published:** 2015-05-28

**Authors:** Joachim Schmutzhard, Peter Lackner, Raimund Helbok, Helene Verena Hurth, Fabian Cedric Aregger, Veronika Muigg, Josua Kegele, Sebastian Bunk, Lukas Oberhammer, Natalie Fischer, Leyla Pinggera, Allan Otieno, Bernards Ogutu, Tsiri Agbenyega, Daniel Ansong, Ayola A. Adegnika, Saadou Issifou, Patrick Zorowka, Sanjeev Krishna, Benjamin Mordmüller, Erich Schmutzhard, Peter Kremsner

**Affiliations:** Department of Otorhinolaryngology, Medical University Innsbruck, Anichstrasse 35, A-6020 Innsbruck, Austria; Department of Neurology, NICU, Medical University Innsbruck, Innsbruck, Austria; Center for Clinical Research, Kenya Medical Research Institute, Kisumu, Kenya; Komfo Anokye Teaching Hospita & Kwame Nkrumah University of Science and Technology, Kumasi, Ghana; Centre de Recherches Médicales de Lambaréné, Albert Schweitzer Hospital (MRUG), Lambaréné, Gabon; Department of Hearing, Speech and Voice Disorders, Medical University, Innsbruck, Austria; St. George’s University of London, London, UK; Institut für Tropenmedizin, Eberhard Karls Universität Tübingen, Tübingen, Germany

**Keywords:** Hearing impairment, Otoacoustic emissions, Severe malaria

## Abstract

**Background:**

Severe malaria may influence inner ear function, although this possibility has not been examined prospectively. In a retrospective analysis, hearing impairment was found in 9 of 23 patients with cerebral malaria. An objective method to quickly evaluate the function of the inner ear are the otoacoustic emissions. Negative transient otoacoustic emissions are associated with a threshold shift of 20 dB and above.

**Methods:**

This prospective multicenter study analyses otoacoustic emissions in patients with severe malaria up to the age of 10 years. In three study sites (Ghana, Gabon, Kenya) 144 patients with severe malaria and 108 control children were included. All malaria patients were treated with parental artesunate.

**Results:**

In the control group, 92.6 % (n = 108, 95 % confidence interval 86.19–6.2 %) passed otoacoustic emission screening. In malaria patients, 58.5 % (n = 94, malaria vs controls *p* < 0.001, 95 % confidence interval 48.4–67.9 %) passed otoacoustic emission screening at the baseline measurement. The value increased to 65.2 % (n = 66, *p* < 0.001, 95 % confidence interval 53.1–75.5 %) at follow up 14–28 days after diagnosis of malaria.

The study population was divided into severe non-cerebral malaria and severe malaria with neurological symptoms (cerebral malaria). Whereas otoacoustic emissions in severe malaria improved to a passing percentage of 72.9 % (n = 48, 95 % confidence interval 59–83.4 %) at follow-up, the patients with cerebral malaria showed a drop in the passing percentage to 33 % (n = 18) 3–7 days after diagnosis. This shows a significant impairment in the cerebral malaria group (*p* = 0.012 at days 3–7, 95 % confidence interval 16.3–56.3 %; *p* = 0.031 at day 14–28, 95 % confidence interval 24.5–66.3 %).

**Conclusion:**

The presented data show that 40 % of children have involvement of the inner ear early in severe malaria. In children, audiological screening after severe malaria infection is not currently recommended, but is worth investigating in larger studies.

## Background

With more than three billion humans at risk of malaria and more than 240 million cases per year worldwide, malaria is one of the most common infectious diseases [1]. Hearing loss affects approximately 278 million people worldwide [2] with approximately two thirds living in resource-poor countries. The high prevalence of hearing impairment in these countries is partly explained by lack of immunization and medical care as well as inadequate funds for intervention once hearing loss has been identified [2]. Hearing impairment associated with malaria has been observed over the past century and has been attributed to the side effects of antimalarials, like quinine [3], chloroquine [4], mefloquine [5], and artemisinin derivates [6, 7]. In an artemisinin combination therapy trial in children aged 0.5–-14 years, uncomplicated malaria has been suspected to be the cause of elevated hearing thresholds. Prior to therapy, hearing thresholds in children with malaria were significantly higher than those seen in the control group [8]. Carter *et al.* suspected severe malaria to be a cause of acquired language disorders [9]. In children, hearing impairment is one of the fundamental causes of language and developmental disorders. Nine out of 23 children with cerebral malaria had impaired hearing in addition to other neurological and cognitive sequelae [10]. So far, no prospective study evaluating hearing with objective tests has been performed for severe malaria.

In a murine model, severe malaria causes significant hearing impairment [11]. Histomorphology revealed an induction of apoptosis in the fibrocytes of the spiral ligament and a breakdown of the blood labyrinth barrier [12, 13]. Both structures are essential to maintain the endocochlear potential, which drives the function of the inner and outer hair cells. The inner hair cells generate the action potential, which contains the hearing information. The outer hair cells are responsible for local amplification of the sound in the cochlea. The contraction of these cells can be measured as otoacoustic emissions (OAE) in the outer ear canal [14].

OAE testing is a specific objective hearing test, which has been used in neonatal hearing screening programs. The presence of OAE confirms a regular function of the inner ear. In transient OAE testing, a click stimulus is applied to the outer ear canal. This stimulus provokes a contraction of the outer hair cells in the inner ear, which can be detected afterwards in the outer ear canal. Transient OAE is generally evaluated as the cross-correlation or “wave reproducibility” of multiple measurements. Wave reproducibility is expressed as a ratio from –100 % to +100 %. A high cross-correlation or wave reproducibility is generally accepted as a measure of normal hearing [15]. Reproducibility of 60 % or below is considered a failed test or impaired hearing. Failed OAE are associated with a hearing impairment of 20 dB or more and require further audiological testing [16]. Transient OAE show a sensitivity of 93 % and a specificity of 67 % in detecting a hearing loss of 30 dB or more [17].

The goal of this prospective multicenter study was to evaluate the inner ear function of children with severe malaria by using transitory evoked OAE and assessing the proportion of children who failed OAE examination.

## Materials and methods

The present study was performed as a sub-study of “Severe Malaria in African Children II” (SMAC) (registered at PACTR201102000277177). The study and the sub-study have been approved by the local ethic committees in Kumasi, Ghana; Kisumu, Kenya; and Lambaréné, Gabon. The studies have been performed in three study sites: Komfo Anokye Teaching Hospital, Kumasi, Ghana; Centre de Recherche Mèdicale de Lambaréné, Lambaréné, Gabon; and Kenya Medical Research Institute, Kisumu, Kenya. Guardian informed consent was obtained from all participants.

### Study design

The study was designed as a prospective multicenter cohort study, comparing OAE measured in children with severe malaria to a healthy, local, age-matched control population. The malaria cohort was divided into a severe malaria group and a cerebral malaria group. OAE were measured in the control group once. In the malaria groups they were measured at the following time points: immediately after diagnosis of severe malaria prior to starting medication, 12–24 h after the first medication, after recovery, 3–7 days post diagnosis, and 14–28 days after the diagnosis. OAE were considered as absent if the reproducibility was below 60 %.

### Cohort selection

The healthy control population was recruited at the study sites outside the hospital in schools and kindergartens. Children aged up to 10 years with no medical history of hearing impairment and no history of fevere related admission to hospital as an in-patient qualified for the control group [18]. No further clinical or laboratory examination was performed.

The severe malaria group was selected according to the following parameters: children up to 10 years; a diagnosis of *Plasmodium falciparum* malaria confirmed with a minimum parasitemia of >5000 parasites/μL on the initial blood smear; asexual forms of *P. falciparum*; and clinical manifestations that required hospitalization, like hyperlactatemia or metabolic acidosis, severe anemia, dark urine, hypoglycemia, jaundice, respiratory distress, severe vomiting, shock, abnormal bleeding, and/or renal failure [19–21]. Antimalarial treatment within 24 h prior to admission was an exclusion criterion.

Cerebral malaria was defined as severe malaria with neurological signs and symptoms like coma with a Blantyre Coma score of ≤ 2, repeated generalized seizures, focal neurological findings, or prostration [21]. Prostration was defined by the presence of one or more of the following symptoms: not being able to breastfeed, sit, stand, or walk depending on the age of the child [20]. Fundoscopy was not done.

For all groups, the ear inspection had to show a transparent tympanic membrane. Children with pathologic findings in the ear canal did not qualify. The baseline OAE testing was done immediately after confirmation of severe or cerebral malaria.

Further clinical and laboratory examinations were performed in the severe malaria and the cerebral malaria group at the time of inclusion in the study. On admission the vital signs—heart rate, temperature, respiratory rate—were documented. The following clinical and laboratory parameters were examined: respiratory distress, deep breathing, severe vomiting, prostration, coma, repeated generalized seizures, jaundice, parasitemia, hemoglobin, platelets, glucose, creatinine, alaninaminotransferase, bilirubin, glucose, hemoglobinuria.

### Malaria therapy

As part of the SMAC II study, all patients were treated with parenteral artesunate after the first baseline measurement. The total dose of artesunate was 12 mg/kg, given as five intramuscular injections of 2.4 mg/kg at 0, 12, 24, 48, and 72 h or as three injections of 4 mg/kg at 0, 24, and 48 h either intramuscularly or intravenously. Allocation to one of the three schedules was random. Upon discharge patients received a weight-adapted standard regimen of artemether and lumefantrine [22].

### Otoacoustic emissions measurement

The OAE measurements were performed at each study site by two operators. Transient OAE were measured using a Madsen Capella Otoacoustic emissions machine (Otometric, Taastup, Denmark). Both ears were measured five times and the best result was taken. If the reproducibility was below 60 % on one ear the OAE were considered absent. Each measurement consisted of 2080 repetitions. A broadband click ranging from 1 kHz to 4 kHz for 2 ms was used as a stimulus. The measurement was considered of a poor quality if the rejection rate exceeded more than 2000 sweeps. Therefore measurements were excluded with more than 2000 rejected sweeps. If the measurement at baseline did not work, the OAE testing was abandoned, and the treatment of the severely sick child immediately initiated. A second try was performed at the other measurement time points.

### Statistical evaluation

Otoacoustic measurements with a reproducibility below 60 % on one ear or both ears were considered a fail. Demographic and clinical data were compared between groups depending on data type and distribution either by Student’s *t*-test or Mann–Whitney *U* test for continuous variables and by chi-square test or Fisher’s exact test for proportions as appropriate. The proportions of patients passing the OAE test were compared between groups (malaria vs control or the different treatment regimens respectively) by chi-square test. The study collective was categorized into severe non-cerebral malaria and cerebral malaria and statistically compared as described above. In order to identify prognostic factors for failing the OAE test at last follow-up, a univariate comparison of admission data was done between patients failing and patients passing on the last follow-up as described above. Variables with association at a significance level of *p* < 0.05 in the univariate analysis were entered into a stepwise logistic regression model with conditional forward selection as the selection method and age and sex as mandatory variables. The statistical evaluation was done by IBM SPSS statistics 21 (New York, NY, USA).

## Results

### Overall results

For the malaria group, 144 patients were recruited and 108 children as controls. Not all patients could be recruited for every measurement, partly because of prior administration of the study medication (baseline, 53 patients), or the patient being absolutely uncooperative (12–24 h, 26 patients, 3–7 days, 20 patients; 14–28 days, 14 patients), or due to loss to follow-up (3–7 days, 12 patients; 14–28 days, 64 patients). No death was to be reported in the study population. The malaria group consisted of 67 female and 73 male patients (four were not documented), the sex distribution in the control group was 50 female and 56 male (two were not documented), with no significant difference in the sex distribution (*p* = 0.898). The mean age in the malaria group was 3.43 years (SD 2.384) and in the control group 3.82 years (SD 2.506).

**Fig. 1:**
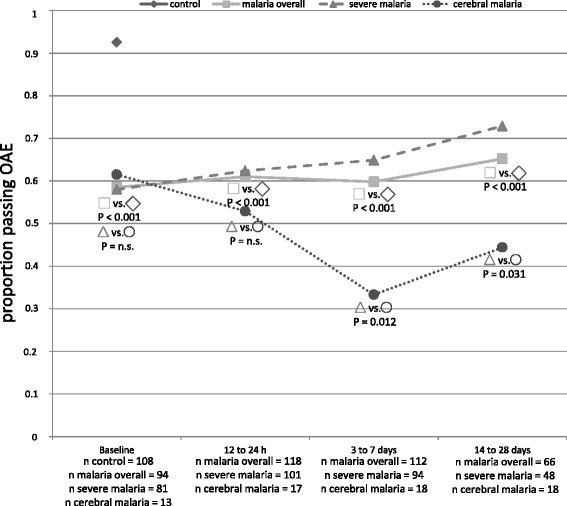
Passing proportions of the OAE. The proportion of passing individuals is shown for the four included groups in the *y-axis* (control group, all malaria patients, severe malaria without neurological symptoms, cerebral malaria). The *x-axis* shows the different measurement time points. The number of included patients for each group is added in the labeling of the x-axis. The *filled symbols* indicate the proportion of passing individuals in each group. The *blank symbols* indicate which figures have been compared for the *p*-values included in the figure

Detailed clinical data for the malaria groups are listed in Table 1. The overall proportion of passes in the control group was 92.6 %. At the baseline measurement only 58 % (*p* < 0.001, 95 % confidence interval (CI), 48.4–67.9 %) of all malaria patients passed the OAE testing. The results of the OAE are listed in Fig. 1. At 12–24 h after prior medication, the overall passing percentage increased to 61 % (95 % CI, 52–69.3 %) with *p* < 0.001 compared to the control group (Fig. 1). At the third measurement time point, 3–7 days after primary medication, the passing percentage was 59.8 % (95 % CI, 50.6–68.4 %; *p* < 0.001; Fig. 1). At the last measurement, 14 to 28 days after inclusion to the study, the average proportion passing was 65.2 % (95 % CI, 53.1–75.5 %) in the malaria group, showing a statistically significant difference at *p* < 0.001 to the healthy control group. No statistical difference of the OAE could be detected between the three different artemether and lumefantrine therapy regimes.

**Table 1:** Clinical data

	Parameter units	Severe malaria		Cerebral malaria		*p*-value
		Result	Mean (SD)	Result	Mean (SD)	Severe malaria vs cerebral malaria
Parasitemia	Parasites/μl	114	97,111 (1,271,985)	24	139,743 (683,264)	NS^d^
Pulse	/min	120	126 (22)	24	132 (21)	NS^c^
Temperature	Celsius	120	38.3 (1.24)	24	37.9 (1.17)	NS^c^
Respiratory rate	/min	120	37.1 (11.5)	24	42.9 (12.3)	0.027^c^
Hemoglobin	g/dl	119	8.7 (2.3)	24	8.3 (2.7)	NS^c^
Platelets	1000/ μl	119	109.310 (94.924)	24	67.458 (50.113)	0.016^d^
White blood cells	1000 /μl	119	9.7 (6.7)	24	10.2 (4.1)	NS^d^
Glucose	mmol/l	98	5.169 (2.342)	20	4.895 (2.521)	NS^c^
Creatinine	g/l	96	23.5 (91)^b^	18	31.5 (568)^b^	NS^d^
Bilirubin	g/l	99	27.5 (184)^b^	21	29 (77)^b^	NS^d^
ALT	U/l	101	32.2 (33.4)	20	34.9 (26.9)	NS^d^
Hemoglobinuria	% of included patients	119	1.7 %	24	4.2 %	NS^e^
Respiratory distress	% of included patients	119	4.2 %	24	12.5 %	NS^f^
Deep breathing	% of included patients	120	1.7 %	24	0 %	NS^f^
Severe vomiting	% of included patients	119	2.5 %	24	4.2 %	NS^f^
Prostration	% of included patients	119	16.8 %	24	54.2 %	0.000^f^
Coma	% of included patients	119	0 %	24	37.5 %	0.000^f^
Repeated generalized seizures	% of included patients	119	0 %	24	50 %	0.000^f^
Jaundice	% of included patients	119	4.2 %	24	12.5 %	NS^f^
Severe anemia	% of included patients	119	10.1 %	24	20.8 %	NS^e^
Hypoglycemia	% of included patients	119	2.5 %	24	8.3 %	0.03^e^

The results of the evaluated clinical data are listed for patients with severe malaria and with cerebral malaria

Mean and standard deviation are given for continuous variables; comparison between groups has been performed by the indicated tests. NS, not significant

^a^Geometric mean (range); ^b^median (range); ^c^Students *t-*test; ^d^Mann–Whitney *U* test; ^e^chi-square test; ^f^Fisher’s exact test

There was no difference in baseline OAE pass rate between the severe malaria without neurological symptoms group and the cerebral malaria group (severe malaria 58 %, 95 % CI, 47.2–68.2 %; cerebral malaria 61.5 %, 95 % CI, 35.5–82.3 %). Within 12 to 24 h, the passing percentage dropped slightly in the cerebral malaria group to 52.9 % (95 % CI, 31–73.8 %) in contrast to a slightly improved passing percentage of 62.4 % (95 % CI, 52.6–71.2 %) in the severe malaria population. The gap further increased at the 3–7 day measurement to a statistical significant value (severe malaria 64.9 %, 95 % CI, 54.8–73.8 %; cerebral malaria 33.3 %, 95 % CI 16.3–56.3 %; *p* = 0.012). A further significant difference was observed at the follow-up 14–28 days after inclusion (severe malaria 72.9 %, 95 % CI, 59–83.4 %; cerebral malaria 44 %, 95 % CI, 24.6–66.3 %) (Fig. 1).

### Prognostic factors for hearing impairment on last follow-up

To identify prognostic factors for hearing impairment at the last follow-up, important demographic, clinical, and laboratory variables were compared between patients passing or failing the last OAE measurement. Reticulocyte count, height, respiratory distress, repeated generalized seizures, and severity group (severe malaria vs cerebral malaria) were associated with hearing impairment at last follow-up. These variables were entered into a stepwise binary logistic regression model with age and sex as mandatory predictors. In this model the significant predictors associated with hearing impairment were age and the malaria severity group. Younger children had a significantly higher risk of hearing impairment (Table 2). Children with cerebral malaria had a 5.09-fold (95 % CI, 1.25–20.77) increased risk for hearing impairment compared to children with severe non-cerebral malaria (Table 2).

**Table 2:** Binary logistic regression model

	Significance	Odd’s ratio	Confidence interval	
			Lower	Upper
Male sex	0.302	2.001	0.536	7.466
Age	0.014	0.728	0.566	0.938
Cerebral malaria	0.023	5.089	1.247	20.771

The results of the stepwise binary logistic regression model are shown, with hearing impairment at the last follow-up as the outcome variable

## Discussion

The present study shows that severe non-cerebral and cerebral malaria [23] may lead to a significant impairment of the inner ear function in the acute stage of the disease. Through the course of the disease the OAE improved in the severe malaria group, whereas there was further impairment of the OAE in the cerebral malaria group.

The control group was recruited outside the hospital with no admission to hospital as an in-patient for fever. Hospitalization for fever had been shown to be associated with significant hearing impairment in sub-Saharan Africa [18]. The malaria group and the control group were well balanced, showing no significant differences for age and sex.

In the control group 92.6 % of the evaluated children passed the otoacoustic testing, indicating a functioning inner ear. An evaluation of preschool children performed in the US showed a similar passing percentage of 91 % in a group comparable for age and sex [24].

In the malaria group the evaluated passing percentage started at 58.5 % at the baseline measurement and increased to 65.2 % at the follow-up 14–28 days after diagnosis, showing highly significant differences. This finding indicates that severe malaria affects the inner ear in 40 % of cases. Transient OAE are absent at a cochlea with hearing loss of 20 dB or more [16]. Similar threshold shifts have recently been reported in uncomplicated malaria by Adjei et al. [8]. Interestingly, the percentage of failures increased through the course of the disease in the cerebral malaria group and decreased in the severe non-cerebral malaria group, showing significant differences at day 3–7 and day 14–28, suggesting that cerebral malaria is more likely to influence cochlear function than severe malaria. The stepwise binary logistic regression model further supported this observation. Children with cerebral malaria showed a 5.09-fold higher risk of OAE failure than children with severe non-cerebral malaria. This observation is supported by a murine malaria study, which showed higher threshold shifts in the cerebral malaria mice and lower threshold shifts in animals with no neurologic symptoms [11].

Cerebral malaria is known to influence the blood–brain barrier [25]. Similar disturbance of the blood labyrinth barrier has been observed in an animal model of cerebral malaria [12]. These pathologic alterations can possibly explain the observed course of the transient OAE. Furthermore, the stepwise binary regression model revealed age as a significant factor for hearing loss. Younger children were more likely to fail transient OAE. This observation can be linked to the fact that young age is a known risk factor for hyperparasitemia with an increased risk of progression to cerebral malaria [26]. Artemisinin ototoxicity has been discussed in various studies with divergent interpretation [7]. Toovey et al. examined the hearing of 150 construction workers with malaria in Mozambique, who were treated with artemisinin-based combination therapies (ACTs). The control group were healthy people without malaria and no ACT treatment. The conclusion of this study was that ACT leads to hearing impairment [27]. Hutagalung et al. performed a comparable study using a malaria-exposed population as control. This study did not reveal any hearing impairment [28]. At the time, their data was interpreted without the knowledge that malaria itself leads to hearing impairment in animal models [11]. Our results suggest that artemisinin does not influence inner ear function in patients with severe malaria, because the recorded otoacoustic impairment was present prior to the first treatment and did gradually improve. Furthermore, the therapy regimes of artemether and lumefantrine were not associated with the otoacoustic emission results. An ototoxic effect of artemisinin would have resulted in a further decrease of the passing percentage.

The statistical evaluation showed a significant association with hearing loss of the following parameters: reticulocyte count, respiratory distress, height, and repeated generalized seizures. Patients with a high count of reticulocytes showed less inner ear impairment than patients with a low count of reticulocytes. This can be explained by the fact that the “anemic malaria” results in an increased count of reticulocytes, but is less likely associated with hearing impairment [12]. This interpretation can also be used for the association of respiratory distress as a result of severe anemia and hearing loss. Respiratory distress is correlated with passing the OAE testing. Height is another factor associated with otologic impairment. Smaller children have an increased risk of hearing loss than taller children. On the one hand this can be linked to the fact that age is a risk factor to develop inner ear impairment, and on the other hand the height effect can be correlated with the nutrition status of the children. A poor nutrition status has been associated with a poor outcome in infectious diseases [29].

A limitation of the study appears to be the lack of tympanometry as an objective evaluation method of the outer ear canal and the middle ear. Lee et al. compared pneumatic otoscopy and tympanometry as tools to diagnose otitis media with effusions. With a sensitivity of 97.2 % otoscopy was superior to tympanometry, which had 87.5 % sensitivity. The gold standard to diagnose otitis media with effusions would be otomicroscopy with a sensitivity of 100 %. Ear microscopy was not available in the study setting. Therefore the better method of the remaining two was used [30]. OAE testing is an objective method to evaluate inner ear function. With a sensitivity of 93 % and a specificity of 67 %, detecting a hearing loss of 30 dB or more is very likely; nevertheless, false positive and false negative results cannot be excluded [17]. Using the identical operators and OAE machine to recruit the control group and the malaria groups suggests an equal failure rate in both groups, thus not influencing the study result. A possible limitation arises from the control group. Healthy children were recruited at the three study sites outside the hospital. A medical history of hearing problems and of hospital admission due to fever, which could be linked to hearing impairment [18], were exclusion criteria for the control group. However, OAE testing in a comparable setting in the US showed an identical passing percentage [24]. A further possible bias was the loss to follow-up of 78 patients, which is more than 50 %. Interestingly, all patients with cerebral malaria patients were included in the last follow-up examination, whereas only 48 patients of the severe malaria group showed up for the last examination. Assuming that individuals in good conditions are more likely to miss a physician appointment, this could be a bias. Nevertheless with all patients with cerebral malaria included, this possible limitation does not account for the cerebral malaria group. A further limitation of the study is the comparably short follow-up period of only 28 days: a long-time effect on the function of the inner ear cannot be reported. Additionally, a further audiologic evaluation of the patients with failed OAE would be necessary to provide final objective evidence of the resulting functional impairment. More studies will be necessary, including a longer follow-up period and additional audiologic evaluations, like auditory evoked brainstem responses. Another limitation of the study is that not all patients could be included at the baseline measurement. When dealing with children who were potentially fatally ill, the primary therapy was not delayed if the OAE team was not ready (e.g. late night admissions). Therefore, quite a number of patients missed the first measurement.

The fundamental pathologic cause of malaria-induced hearing loss is not fully understood. Severe malaria animal models have detected induction of apoptosis in the fibrocytes of the spiral ligament [12], in which the blood labyrinth barrier is positioned. Evans blue staining of the “malaria infected cochlea” has shown a breakdown of the blood labyrinth barrier, influencing the electrolyte circulation of the cochlea [12]. The fact that cerebral malaria is more likely to cause failure of OAE might be explained by the elevated intracranial pressure associated with cerebral malaria [31]. Elevated pressure to the cochlea, originating from the intracranial space has been suspected to cause cochlea damage [32]. Vice versa, the release of intracranial pressure has been associated with an improvement of the OAE [33]. Further in vivo animal studies are required.

## Conclusion

The present data show that severe malaria and cerebral malaria lead to a cochlea malfunction of 20 dB or more in > 40 % of patients. With 240 million cases per year, only a small percentage of individuals with persistent hearing impairment still results in a huge number of disabled individuals, with enormous developmental and socioeconomic impact [34]. These data should increase awareness that malaria may lead to hearing impairment in children. Hearing screening should become a standard examination after malaria infection in children.

## Abbreviations

ACT: Artemisinin combination therapy
OAE: Otoacoustic emissions
SMAC: Severe Malaria in African Children
