# Erratum to: Severe malaria in children leads to a significant impairment of transitory otoacoustic emissions - a prospective multicenter cohort study

**DOI:** 10.1186/s12916-016-0616-4

**Published:** 2016-04-22

**Authors:** Joachim Schmutzhard, Peter Lackner, Raimund Helbok, Helene Verena Hurth, Fabian Cedric Aregger, Veronika Muigg, Josua Kegele, Sebastian Bunk, Lukas Oberhammer, Natalie Fischer, Leyla Pinggera, Allan Otieno, Bernards Ogutu, Tsiri Agbenyega, Daniel Ansong, Ayola A. Adegnika, Saadou Issifou, Patrick Zorowka, Sanjeev Krishna, Benjamin Mordmüller, Erich Schmutzhard, Peter Kremsner

**Affiliations:** Department of Otorhinolaryngology, Medical University Innsbruck, Anichstrasse 35, Innsbruck, A-6020 Austria; Department of Neurology, NICU, Medical University Innsbruck, Innsbruck, Austria; Center for Clinical Research, Kenya Medical Research Institute, Kisumu, Kenya; Komfo Anokye Teaching Hospita & Kwame Nkrumah University of Science and Technology, Kumasi, Ghana; Centre de Recherches Médicales de Lambaréné, Albert Schweitzer Hospital (MRUG), Lambaréné, Gabon; Department of Hearing, Speech and Voice Disorders, Medical University, Innsbruck, Austria; St. George’s University of London, London, UK; Institut für Tropenmedizin, Eberhard Karls Universität Tübingen, Tübingen, Germany

## Erratum

After publication of the original article [1], it came to the authors’ attention that a source of funding for the clinical trial was inadvertently omitted from the Acknowledgement section. The European and Developing Countries Clinical Trials Partnership (EDCTP) should have been mentioned as a part funder, so the Acknowledgement section should have read as follows:
